# Silent speech command word recognition using stepped frequency continuous wave radar

**DOI:** 10.1038/s41598-022-07842-9

**Published:** 2022-03-09

**Authors:** Christoph Wagner, Petr Schaffer, Pouriya Amini Digehsara, Michael Bärhold, Dirk Plettemeier, Peter Birkholz

**Affiliations:** 1 Institute of Acoustics and Speech Communication, Chair for Speech Technology and Cognitive Systems, Technische Universität Dresden, 01069 Dresden, Germany; 2 Institute of Communication Technology, Chair of Radio Frequency and Photonics Engineering, Technische Universität Dresden, 01069 Dresden, Germany

**Keywords:** Electrical and electronic engineering, Biomedical engineering

## Abstract

Recovering speech in the absence of the acoustic speech signal itself, i.e., *silent speech*, holds great potential for restoring or enhancing oral communication in those who lost it. Radar is a relatively unexplored silent speech sensing modality, even though it has the advantage of being fully non-invasive. We therefore built a custom stepped frequency continuous wave radar hardware to measure the changes in the transmission spectra during speech between three antennas, located on both cheeks and the chin with a measurement update rate of 100 Hz. We then recorded a command word corpus of 40 phonetically balanced, two-syllable German words and the German digits zero to nine for two individual speakers and evaluated both the speaker-dependent multi-session and inter-session recognition accuracies on this 50-word corpus using a bidirectional long-short term memory network. We obtained recognition accuracies of 99.17% and 88.87% for the speaker-dependent multi-session and inter-session accuracy, respectively. These results show that the transmission spectra are very well suited to discriminate individual words from one another, even across different sessions, which is one of the key challenges for fully non-invasive silent speech interfaces.

## Introduction

Silent speech interfaces (SSI) have received considerable attention over the last decade^1–3^. All of them have in common that they aim at restoring or enhancing oral communication from coexistent, non-audible (bio)signals which are generated during speech production, even in the absence of the acoustic speech signal itself. Their potential applications range from voice restoration for patients who underwent laryngectomy^4,5^ to enabling private conversations in public areas and enhancing speech intelligibility in noisy environments^3^. For this purpose, a number of measuring modalities have been proposed that differ with respect to the type of biosignal they leverage, and whether these signals can be measured invasively or non-invasively.

Invasive measuring techniques include permanent magnetic articulography (PMA)^4,6–8^, electromagnetic articulography (EMA)^9–12^, electropalatography (EPG)^13,14^ and electro-optical stomatography (EOS)^15,16^, a combination of EPG and optopalatography^17^. Whereas only implant-based PMA is truly invasive, the remaining techniques require at least some components of the measuring device to reside inside the oral cavity, either by attaching magnets or coils to the tongue and lips (PMA, EMA) or by placing the sensing device against the hard palate (EPG, OPG, EOS).

Non-invasive measuring techniques are surface electromyography (sEMG)^18–22^, ultrasound (US) doppler^23^, US imaging^24^, video imaging^25,26^ (or a combination of both^27^) and radar-based sensing (RBS)^28–34^. For sEMG, the electrodes are placed on specific locations on the neck, face and chin (above the muscles involved in speech production^21^), whereas for US, the ultrasound probe is either placed below the chin, facing upwards, to capture tongue movements^27^, or in front of the mouth^23^. Video imaging captures the lips and for RBS, one or several antennas are placed either on the facial skin^31^ or in front of the mouth, as well^28–30^.

Especially sEMG and RBS have a number of advantages over the other methods: their non-invasive nature is naturally more appealing to a broader range of potential users and does not impair speech movements as much (or at all). The sensing probes (electrodes or antennas) can be made relatively small and are light-weight, while the size constraints are not as rigorous as compared to intraoral sensors, which need to be as small as possible^17^. They can also be placed on, or in very close proximity to the skin, which is more difficult for, e.g., the proposed video imaging based lip reading methods. Given the body of literature and its recent advances in large vocabulary recognition^21^, sEMG is currently the most developed measuring technique for SSIs. sEMG requires the use of secondary articulatory signals (i.e., the muscle’s electrical surface potential) as opposed to direct measurement techniques like US, EMA, PMA, OPG or EOS, which capture the actual location of the tongue (or part of it) and lips in 2D or 3D space. As a result, sEMG has historically suffered from high signal variability when the electrodes were replaced or moved slightly. Several methods have been proposed to reduce this so called *inter-session* variability substantially^35^, but it remains an intrinsic difficulty of SSIs that use sensors which can vary in their placement.

In contrast to sEMG, radar-based SSIs are still largely unexplored, although the idea has been around for over 20 years^28^. Additionally, most work has focused on inferring articulator movements from *reflected* electromagnetic waves (effectively a form of *remote sensing*) with antennas placed in front of visible articulators (without contact), instead of placing the antennas directly on the skin. In that manner, Holzrichter *et al.* performed qualitative interferometric measurements of the reflected signals from mouth, larynx and glottis with a continuous wave (CW) radar at a frequency of 2.3 GHz^28,36^. Eid and Wallace used ultra-wideband impulse radar (UWB-IR) to measure the reflection coefficient $\Gamma$ ($S_{11}$) with a frequency range of around 3-10 GHz^29^ and conducted a proof-of-principle recognition experiment on ten english digits “zero” to “nine”. Shin *et al.* also used UWB-IR to interferometrically measure the frequency-dependent displacement between the radar sensor and the mouth & lips, expanding the same ten digit corpus to five English vowels^30^. Wen *et al.* presented qualitative CW interferometric displacement measurements during speech with a frequency of 120 GHz, but without a follow-up recognition experiment^33^. Only recently Birkholz *et al.* demonstrated that both the reflection and *transmission* spectrum of antennas placed directly on the facial skin also carry valuable information about the vocal tract shape to distinguish a set of 25 German context-dependent vowels and consonants from one another^31^. The measurements were recorded with a conventional spectrum analyzer, however, which limited the acquisition speed for a full sweep to around 2.7 Hz, which is too slow to measure speech in real-time.

Building on these results, we developed a custom acquisition hardware (introduced in the next Section), capable of measuring the transmission spectra through the vocal tract with a measurement update rate of at least 100 Hz, which is usually set as the lower bound for real-time speech acquisition in SSIs. To evaluate the potential of inferring silently uttered speech, we conducted an offline isolated word recognition experiment and determined both the speaker-dependent multi-session and inter-session recognition accuracies. We conclude with a detailed discussion on our findings.

## Methods

### General system requirements, antennas and placement

**Figure 1:**
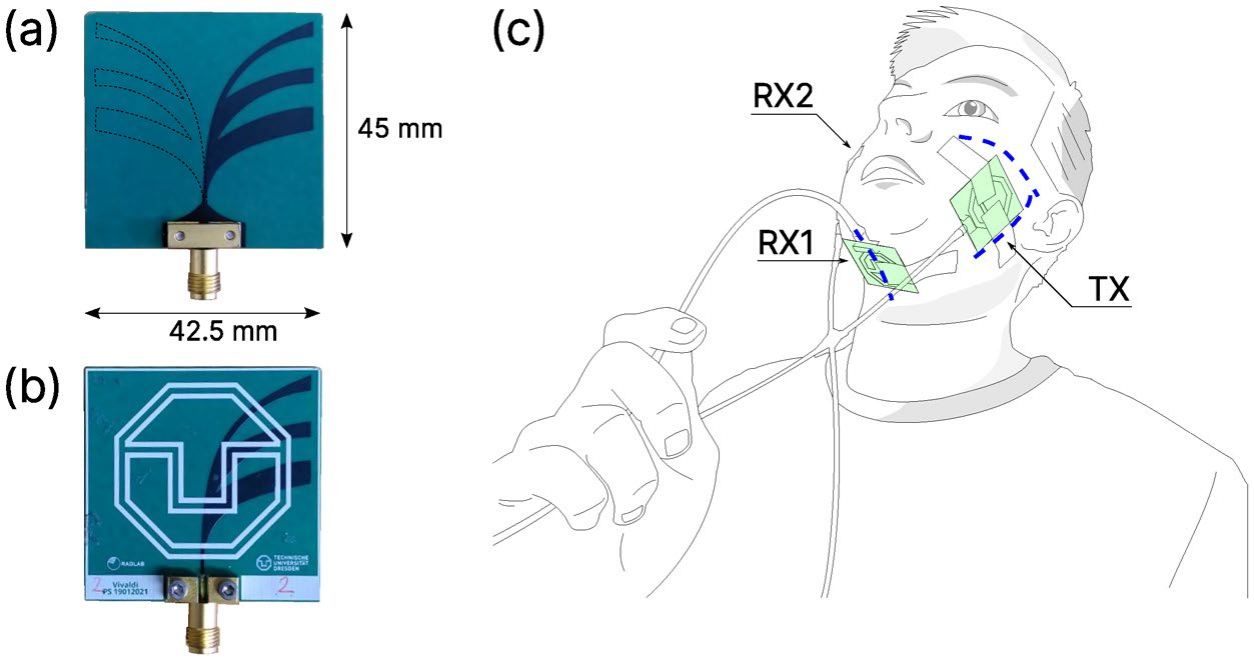
(**a**) Display of the used Vivaldi antenna’s side oriented towards the skin and (**b**) away from the skin. Dashed lines indicate the location of the two diametrically opposite antenna parts (**c**) Depiction of the location of the three antennas as placed on the cheeks and chin. The non-visible antenna for port RX2 on the right cheek is placed exactly like the left cheek’s TX antenna. Dashed blue lines mark the zygomatic bone, part of the mandibula and the chin’s center line, all of which served as landmarks during placement.

To reach the required measurement update rate of 100 Hz, the radar hardware needed to measure a single sweep for all input channels in less than 10 ms. This frame rate is necessary to capture short phonemes sufficiently well. The frequency range was set to 1–6 GHz. The lower bound (1 GHz) was primarily limited by the selected frequency mixer, whereas the upper bound was chosen because of high attenuation below the device’s noise floor observed above this frequency. The selected antenna type was an antipodal Vivaldi antenna, very similar to the one used in our previous study^31^, except for a more rigid substrate. Three antennas were placed on the left and right cheek and on the chin (Fig. 1). The antenna on the left cheek was always the sending (TX) antenna, whereas the right cheek’s antenna was always the receiving port 2 antenna (RX2), and the chin’s antenna the receiving port 1 antenna (RX1). As such, both antennas measured the transmission of the electromagnetic waves through the vocal tract for two different locations and orientations. All antennas were fixated to their location with a double-sided medical-grade skin adhesive tape (type 1510, 3M) and their edges further fixated with a single-sided medical-grade tape.

### System design and architecture

**Figure 2:**
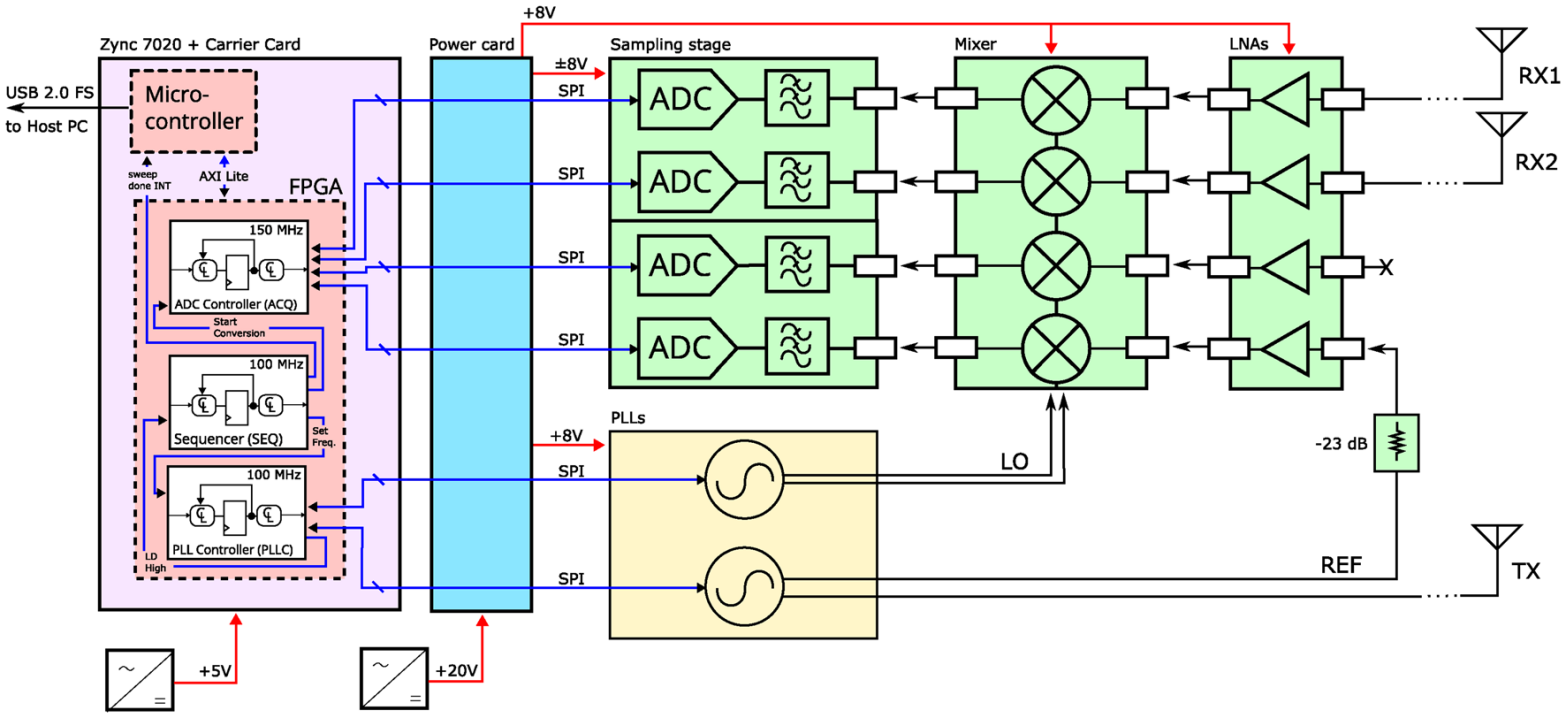
Block diagram of the SFCW radar system, including the antennas. Power-, control-, and measurement signals are displayed as red, blue and black connections, respectively.

**Figure 3:**
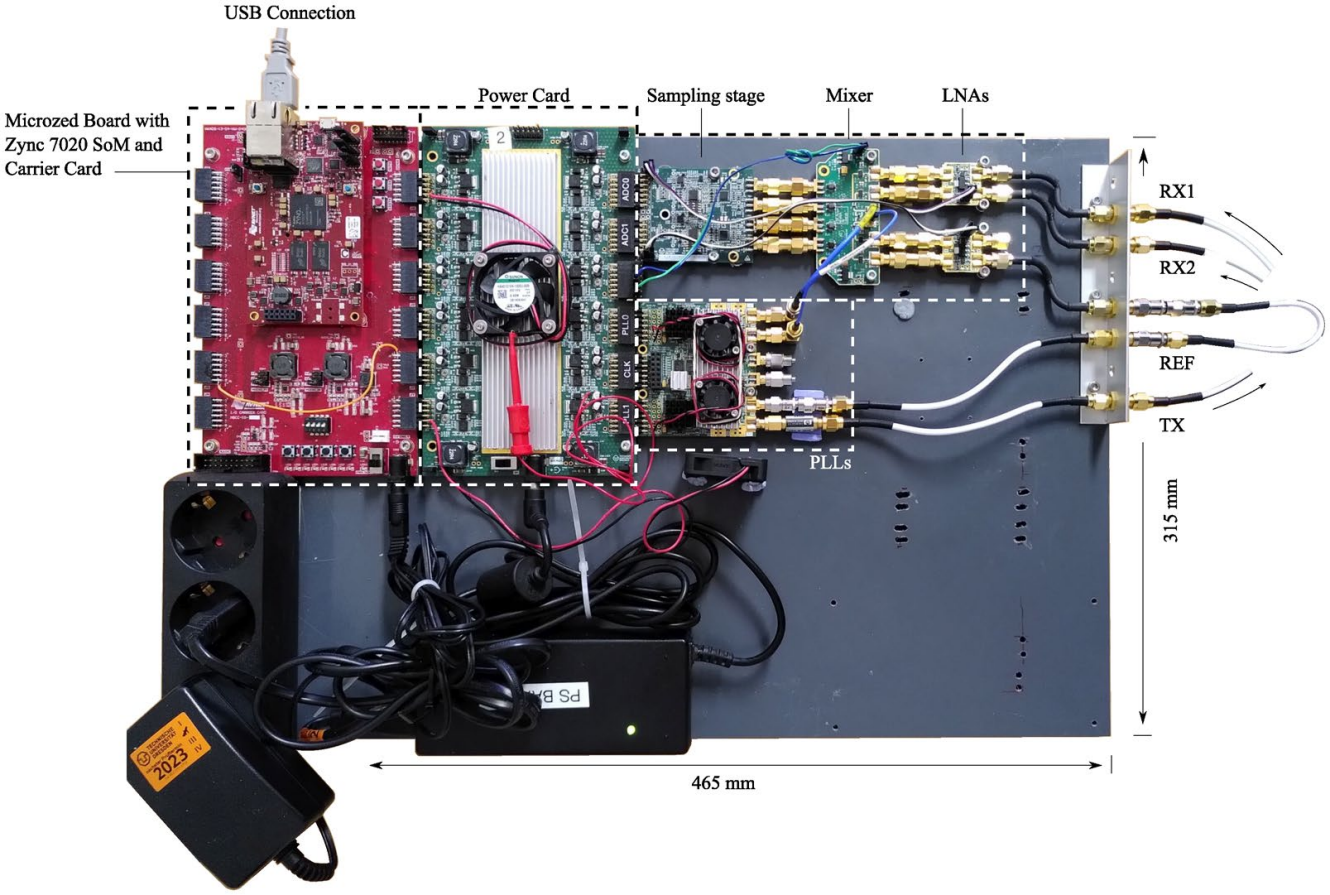
Actual hardware implementation according to the block diagram depicted in Fig. 2 (excluding the antennas).

The measuring system was a stepped frequency continuous wave (SFCW) radar with a 2 (+1) heterodyne architecture, i.e., there were two receiving signal ports and a reference signal port. A block diagram of the hardware implementation is displayed in Fig. 2. The corresponding hardware prototype is displayed in Fig. 3.

The receiver part of the system consisted of the four independent inputs with two of them connected to a receiving antenna (RX1 and RX2) and one driven by a reference signal (REF) (and one spare input for future use). Each input channel implemented a low-noise amplifier, a downmixer stage to convert the radio-frequency (RF) signal down to a fixed intermediate frequency (IF) $f_{\mathrm {IF}}$ of 1 MHz, a low-pass filter (type LTC6603, Linear Technology) with its programmable cut-off frequency and gain set to 1.407 MHz and 24 dB, respectively, and a final sampling stage with two 14-bit dual-channel SAR ADCs. The RF transmitter frontend used two wideband RF synthesizers (type LMX2594, Texas Instrument) to generate two frequencies with a predefined offset over the entire system bandwidth. Using both outputs of the two synthesizers provided 4 signals in total, labelled LO (2x), TX and REF. The output level of the TX synthesizer were set to the part’s specific, dimensionless minimal power level of 0, whereas the power level of the LO synthesizer was set to 50, resulting in an approximately 6-7 dBm maximal output power according to the devices’ datasheet. The REF signal was attenuated by -23 dB to prevent clipping, since both the REF and TX signals were generated from the same synthesizer with the same output power level, but the TX signal would experience significantly more attenuation through the vocal tract. The power card block supplied all modules from a single +20 $\text {V}_{\text {DC}}$ power supply. DC/DC conversion for the various components of each module was done on the respective module itself and left out for clarity. A MicroZed development board (containing a Xilinx Zync7020 SoM) in combination with a MicroZed I/O Carrier Card was used to control the radar system. Sequencing (SEQ) control and signal acquisition (ACQ) was implemented on the FPGA in separate logic modules, whereas baseband conversion and communication with the host PC was realized on the embedded ARM microcontroller. The ACQ module was clocked with 150 MHz, which was necessary to reach the ADC’s sampling rate of 4 MHz, whereas all other modules were clocked with 100 MHz. A set of SCPI commands were used to control the hardware device via the host PC, e.g., to start the acquisition or to change the frequency spacing of the sweep.

### Measuring routine

The measuring routine for signal acquisition and data transmission is summarized as follows: To start the measurement, the respective SCPI command was sent to the device which subsequently activated the SEQ module. The SEQ module then set both PLL frequencies $f_n^{\mathrm {(LO)}}$ and $f_n^{\mathrm {(TX)}} = f_n^{\mathrm {(LO)}} + f_{\mathrm {IF}}$ for $n = 1,2,\dots , N$ frequency steps sequentially over SPI. For each frequency step *n*, once the SEQ module received a valid lock detect signal from both synthesizers (signaling a stable frequency output), it strobed the ACQ module *K* times (where *K* is the number of samples per frequency step) to acquire all $K\times 3$ ADC samples for all three channels simultaneously at the 4 MHz sampling rate and stored them in a FIFO on the FPGA. After all *N* frequency steps, the SEQ module triggered an interrupt to signal the finished acquisition. The FIFOs, containing $K\times N\times 3$ ADC samples, were read out via an AXI Lite interface, converted to baseband according to Eqs. (1)–(3) and stored in system memory. When finished, a single *frame* (containing the two transmission spectra $S_1(f)$ and $S_2(f)$) was sent to the host PC. The real and imaginary parts of each complex frequency component $S_{1,2}(f_n)$ were transmitted via USB 2.0 FS as a Q1.14 16-bit signed integer and converted to a 32 bit float value on the host side.

Because the IF signals $x_{f_n}(t)$ were sampled with four times the IF signal frequency (at 4 MHz) the real and imaginary parts of each complex frequency component $X_{f_n}$ were calculated for every 4 consecutive signal samples $x[0],\dots , x[3]$ at the corresponding RF $f_n$ according to

1 $$\begin{aligned}&\mathrm {Re}\{X_{f_n}\} = x_{f_n}[0] - x_{f_n}[2] \end{aligned}$$

2 $$\begin{aligned}&\mathrm {Im}\{X_{f_n}\} = x_{f_n}[1] - x_{f_n}[3]. \end{aligned}$$

The resulting complex frequency components $S_{1}(f_n)$ and $S_{2}(f_n)$ of the transmission spectra $S_{1}(f)$ and $S_{2}(f)$ at the discrete frequencies $f_n,\,\, n \in [1,2,\dots ,N]$ were then calculated as

3 $$\begin{aligned} S_{1,2}(f_n) = \frac{X_{1,2}(f_n)}{X_{\mathrm {Ref}}(f_n)}. \end{aligned}$$

To increase SNR, all signals were $4\times$ oversampled ($OS=4$) and simply averaged (equivalent to filtering with a rectangular window function). As such, for every discrete frequency step, $K=4\times OS\times 3$ samples were acquired (a total of 16 samples for each of the 3 channels).

### Study design and data acquisition

The command word corpus consisted of 40 phonetically balanced, two-syllable German words and the German digits zero to nine. Its content is summarized in Table 1.

**Table 1:** Set of command words along with their IPA transcription.

Null	(/nʊl/)	Jury	(ʒʏʁiː/)	Neubau	(/nɔybau /)
Eins	(/ains/)	Mörser	(/mœɐzɐ/)	Judo	(/juːdo/)
Zwei	(/ʦvaɪ/)	Wohnhaus	(/voːnhaʊs/)	Regie	(/ʁeʃiː/)
Drei	(/dʁaɪ/)	Ketchup	(/kɛtʃʊp/)	Akku	(/akʊ/)
Vier	(/fiːɐ/)	Feuer	(/fɔʏɐ/)	Böschung	(/bœʃʊŋ/)
Fünf	(/fʏnf/)	Büro	(/bʏʁoː/)	Hausmüll	(/haʊsmʏl/)
Sechs	(/zɛks/)	Detail	(/detaːɪ/)	Vision	(/viːZ̭joːn/)
Sieben	(/ziːbṇ/)	Juli	(/juːlɪ/)	Zeugin	(/ʦɔʏgɪn/)
Acht	(/axt/)	Ehe	(/eːə/)	Depot	(/deːpoː/)
Neun	(/nɔyn/)	Shampoo	(/ʃampu/)	Buffet	(/bʏfeː/)
Dachstuhl	(daxʃtuːl)	Höhe	(/høːə/)	Duell	(/duːɛl/)
Nähe	(/nɛːə /)	Gage	(/gaʒɛ/)	Nachteil	(/naxtaɪl/)
Feier	(/fai ɐ/)	Züchtung	(/ʦʏçtʊŋ/)	Mühe	(/myːə/)
böig	(/bøːɪç/, /bøːɪk)	Cello	(/ʦɛoː/)	Ära	(/ɛːʁa/)
Ziehung	(/ʦiːʊŋ/)	Wiese	(/viːzə/)	Hirschkuh	(/hɪʁʃku/)
Chaos	(/kaːɔs/)	fähig	(/fɛɪç/, /fɛɪk/)	Lösung	(/løzʊŋ/)
Ego	(/eːgɔ/)	Locher	(/lɔxɐ/)		

**Figure 4:**
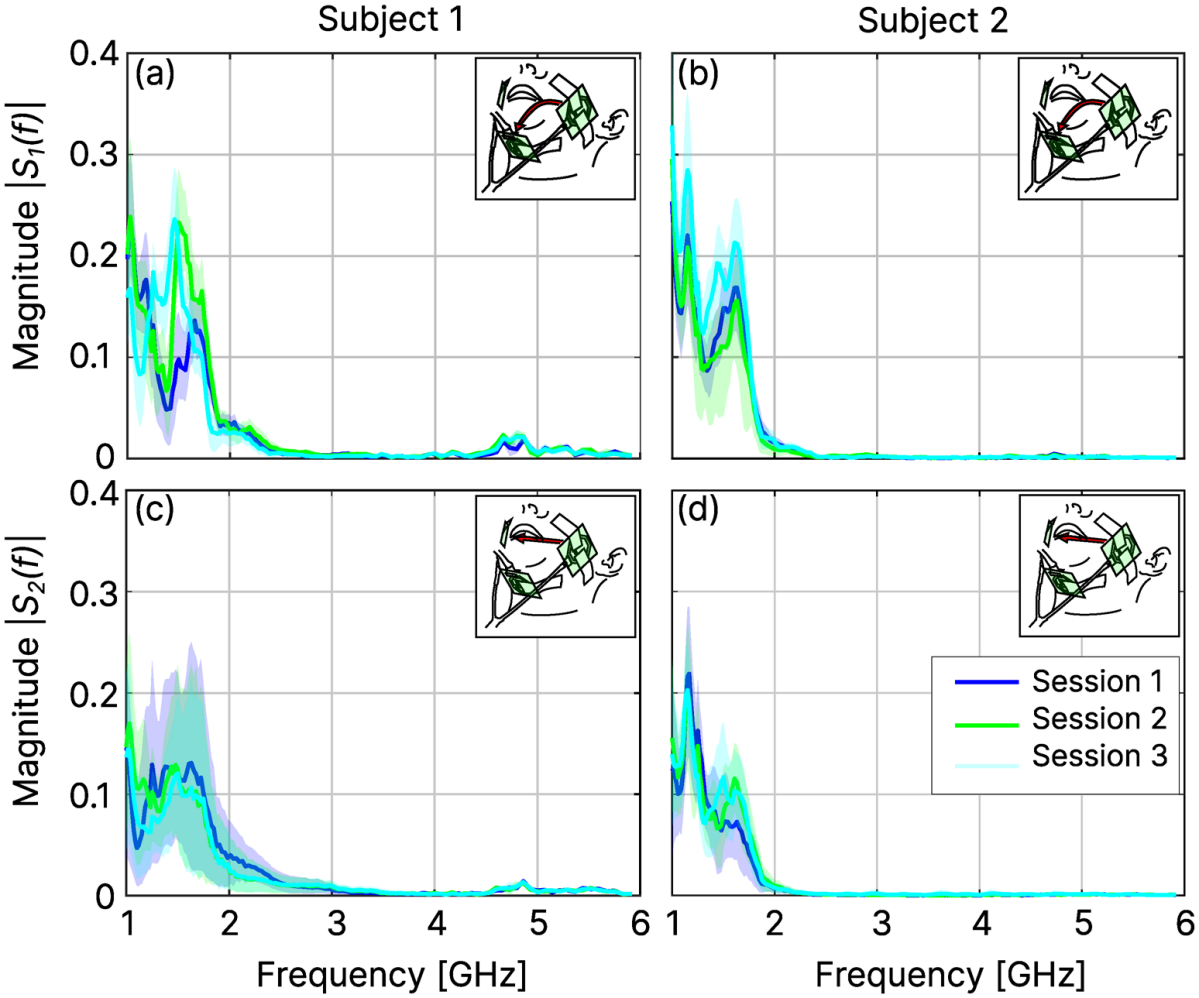
LTAS of the transmission spectra (solid lines) for the cheek-to-chin (**a**, **b**) and cheek-to-cheek path (**c**, **d**) for both subjects and all three sessions. Shaded areas display the [5%, 95%] quantiles around each frequency point.

A custom C++ graphical user interface (using the framework wxWidgets 3.1.3) was written to control the device, record the data, inspect them in real-time, and manually segment the recorded spectrograms after acquisition. Both the audio and radar data streams were recorded simultaneously with a fixed sample rate of 44100 Hz and 100 Hz, respectively. Each transmission spectrum was measured at $N=128$ discrete frequency points, logarithmically spaced between 1 and 6 GHz. The choice of 128 frequency points was a compromise between spectral resolution and a stable measurement speed of 100 Hz. The logarithmic spacing was chosen due to the increase in attenuation towards higher frequencies (see Fig. 4) and thus to increase the spectral resolution for lower frequencies, while still covering the full frequency band up to 6 GHz.

Recordings were taken from two native German male subjects (age 32 and 36) with a total of three sessions each, over the course of two days in a quiet office room. Both subjects remained seated and faced the same direction during the recordings. Informed consent was obtained from both participants after a thorough explanation of the experimental procedure. The experiment was approved by the Ethical Board Committee of the Technische Universität Dresden (approved 9.11.2021, protocol number SR-EK-486102021), conducted in accordance to the principles of the Declaration of Helsinki and following relevant German guidelines and regulations. During each session, every word in Table 1 was recorded (vocalized) one after another (column-wise), ten times in a row for a total of 500 utterances per session and 10 repetitions of each word. The full command word corpus thus consisted of $3\times 500$ utterances per subject, for a grand total of 3000 utterances. To be able to test for inter-session variability, all three antennas were removed after each session, the adhesive tape replaced and then reattached to approximately the same location, checked only by visual inspection.

The recorded radar spectrograms were manually endpointed into the individual utterances (words) on the basis of the audio data. Start and end marks were set at approximately 50 ms (equal to 5 radar frames) before and after each utterance. Each utterance was saved as three individual files, containing the spectrograms (custom binary format), the audio data (as .wav files) and the word label (as plain .txt files).

### Classifier

Isolated word recognition presents a sequence-to-label task, in which the input sequence has to be mapped to one of the possible word labels in the vocabulary. A number of different mapping approaches have been used for this purpose and in the context of silent speech recognition, such as Hidden Markov Models (HMMs) paired with Gaussian Mixture Models (GMMs)^7,19^ support vector machines^11^, linear discriminant analysis^22^, dynamic time warping (DTW) or template matching with k-nearest neighbors (kNN)^6,29^, classical feed-forward neural networks^18,24^ and more recently, recurrent neural networks (RNNs), specifically long-short term memory networks (LSTMs)^12^. In this study, a bidirectional LSTM (BiLSTM), was selected as the classifier of choice. The full network architecture was kept very simple and comprised of the input layer, followed by a single BiLSTM layer, a fully connected layer and a softmax layer. The implementation was done with the PyTorch framework^37^. The following parametrization was used: learning rate $\in$ [$5\cdot 10^{-3}$, $5\cdot 10^{-2}$], number of hidden units $\in$ [20, 100] and batch size of 8. The maximal number of training epochs was set to 200, but was never actually reached due to *early stopping* (with patience of 20), based on the validation sets’ accuracy evaluated after every epoch.

### Feature sets

Whereas both transmission spectra were recorded for frequencies from 1 to 6 GHz, only the frequencies from 1 to 2.5 GHz (equivalent to the first 67 frequency points) were found to actually contain any meaningful spectral energy and were thus considered as input features. Overall, six different sets of features were evaluated:

1. spectral magnitude of $S_{1}(f)$ (67 features)
2. spectral magnitude of $S_{2}(f)$ (67 features)
3. spectral magnitude of $S_{1}(f)$ and $S_{2}(f)$ (134 features)
4. spectral magnitude of $S_{2}(f)$ and $\Delta S_{2}(f)$ (134 features)
5. spectral magnitude of $S_{2}(f)$ and $\Delta S_{2}(f)$ & phase of $\Delta S_{2}(f)$ (201 features)
6. spectral magnitude of $S_{1}(f)$, $S_{2}(f)$, $\Delta S_{1}(f)$ and $\Delta S_{2}(f)$ (268 features).

In all cases, linear magnitudes were used (no scaling to decibel). $\Delta S_{x}(f)$ are *delta* features, calculated as the difference $S_x^{(t+\Delta t)}(f_n) - S_x^{(t)}(f_n)$ of two adjacent complex spectra^7,29^. Finally, all features were normalized to [0,1] on a per-subset basis, e.g., for set 3, $S_{1}(f)$ and $S_{2}(f)$ were individually normalized.

### Evaluation procedure

For reasons of comparability with word recognition results from other SSI approaches, we tested the speaker-dependent *multi-session* and *inter-session* recognition accuracy of our system^2,35^.

For the multi-session evaluation and for each of the two subjects individually, all sequences from all three sessions were combined and subsequently split into a stratified, randomly sampled training, validation, and hold-out test set in a 70%/10%/20% ratio (21, 3 and 6 repetitions per word), respectively. As such, the classifier was trained on 1200 sequences and tested on the remaining 300 and instances of all sessions were present in the training and test set.

In case of the inter-session evaluation, a total of three classification experiments were evaluated per subject, where two of the three sessions were used as the training set, whereas the third session was left out as the hold-out test set, resulting in three {training set | test set} splits: {session 1, session 2 | session 3}, {session 1, session 3 | session 2}, {session 3, session 2 | session 1}. Technically, this can also be regarded as a form of multi-session, however, there was always one session which was not seen by the classifier during training (in the sEMG literature, this is also termed *session combination*^35,38^).

A stratified, randomly sampled subset containing 20% of the training sets’ sequences (i.e., 4 sequences for each class) was used as the validation set, whereas the remaining 80% (with 16 sequences for each class) were used for training. For both the multi-session and inter-session classification experiments, hyperparameter optimization was done with respect to the highest accuracy on the validation set without cross-validation and for a total of 20 randomly sampled hyperparameter sets. The test set was subsequently evaluated on the network with the optimal set of hyperparameters. In several cases, the hyperparameter search did not reveal the optimal set of hyperparameters, i.e., a different set of hyperparameters would have lead to a higher test accuracy. In those cases, the sub-optimal test accuracy was reported nonetheless. Because of this observation, a follow-up experiment was conducted, where the validation set was not taken from the training set (the first two sessions), but as a 20% subset from the test set (the remaining session). This could be considered as a form of “hyperparameter finetuning” (or a weak form of *session adaptation*^35^).

## Results

Figure 4 shows the long-term average transmission spectra (LTAS) for both transmission paths (cheek to cheek ($S_2(f)$) and cheek to chin ($S_1(f)$)) and both subjects 1 & 2, calculated across all sequences of a single session, to provide a sense of the spectral dynamics for each frequency and for the overall spectral similarity between subsequent sessions. Whereas both transmission spectra were heavily damped from 2-2.5 GHz onwards, $S_{2}(f)$ showed substantially more dynamic for both subjects, especially for subject 1, as well as more consistency across sessions.

**Figure 5:**
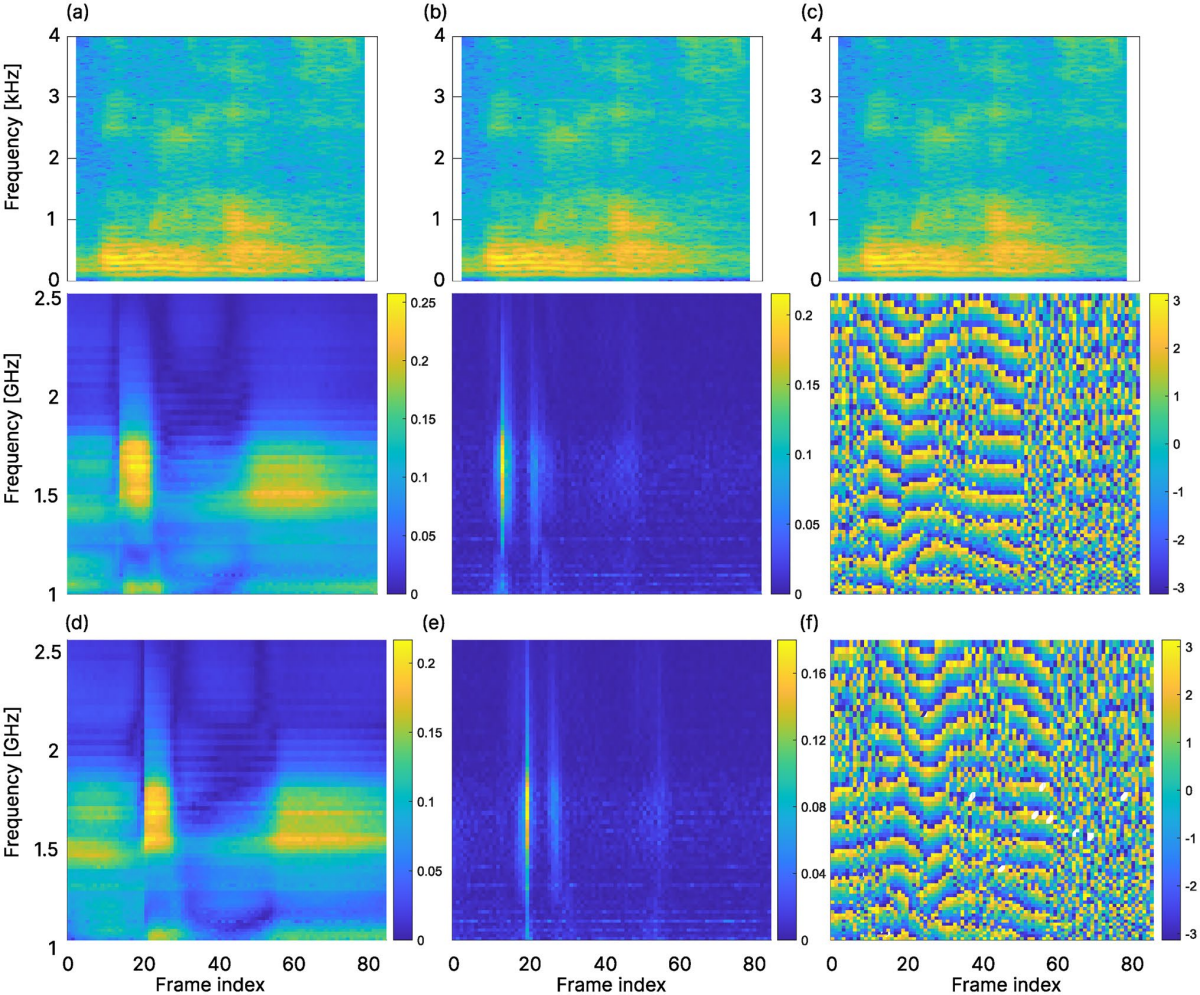
Frame-aligned audio spectrogram (top row, 2048 point FFT, hann-windowed, 1536 samples overlap) and radar spectrogram (middle and bottom row) of the exemplary word “wohnhaus” (/voːnhaʊs/) for the selected frequency range from 1 to 2.5 GHz. (**a**): Linear magnitude radar spectrogram $|S_2(f)|$ (84 frames). (**b**, **c**): corresponding magnitude and phase delta features, respectively. (**d**–**f**): $|S_2(f)|$ and delta features for another repetition from a different session of the same word (88 frames).

**Figure 6:**
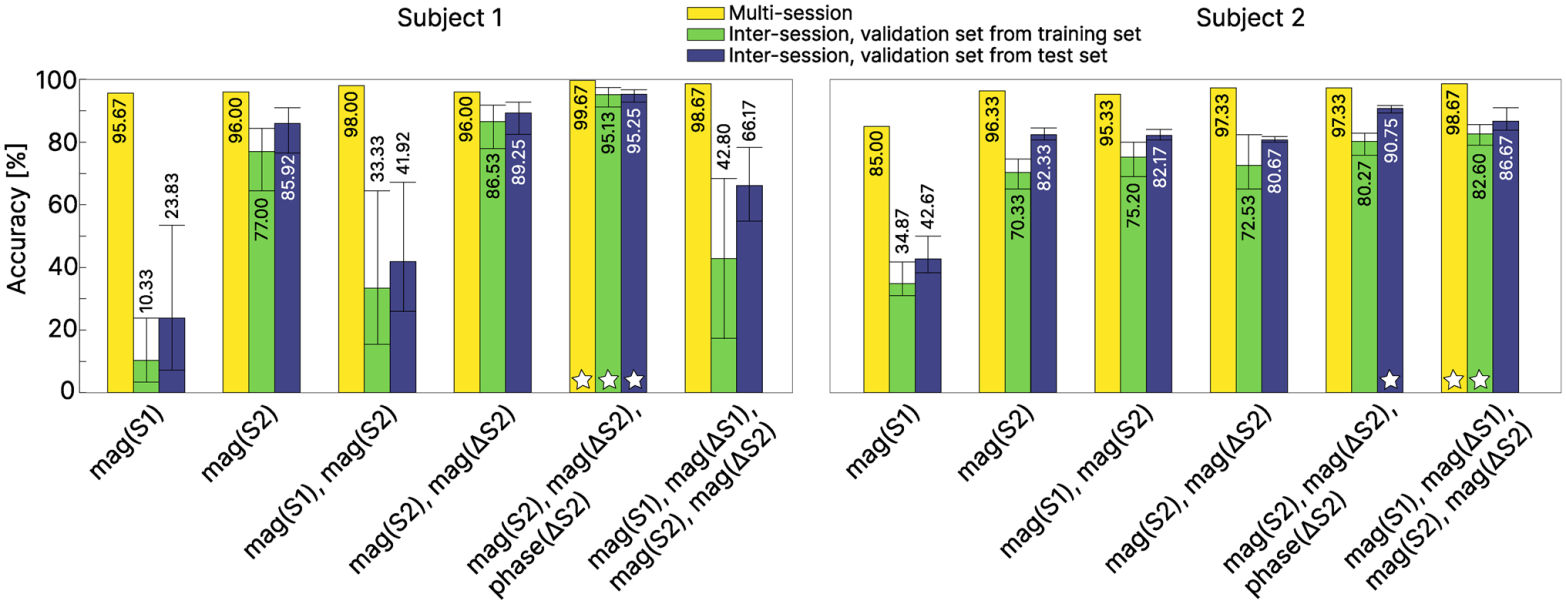
Multi-session and inter-session results for both subjects, sorted by feature sets. The inter-session results are reported with their mean, minimal and maximal value of the three individual splits combined. Values are displayed for the respective mean value. A white star ($\star$) indicates the best-performing feature set in each category.

**Figure 7:**
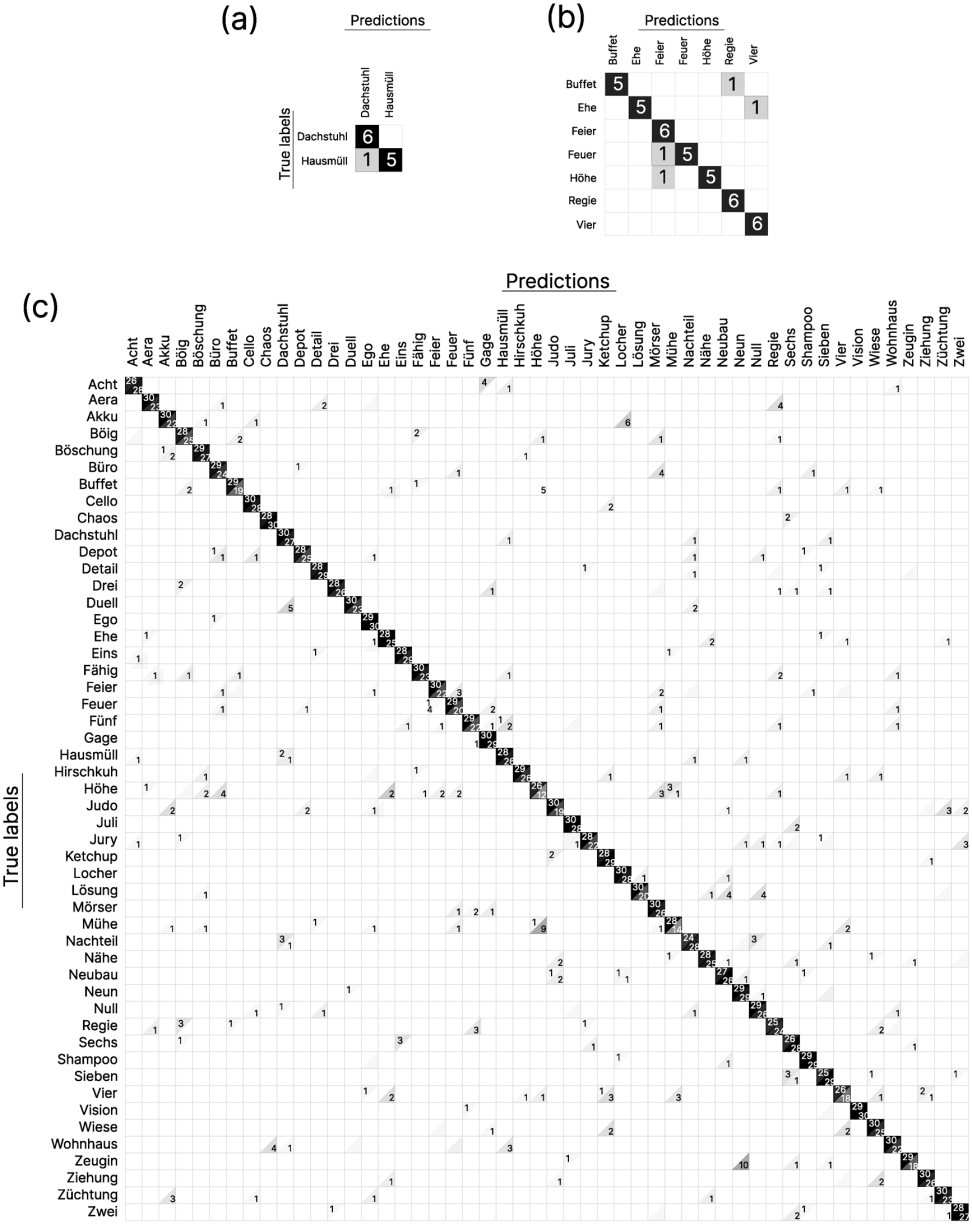
Confusion matrices for all recognition experiments. (**a**) multi-session, subject 1. (**b**) multi-session, subject 2. Rows with perfect classification results are omitted here for clarity and brevity. (**c**) inter-session, subject 1 and 2 combined (upper and lower triangles, respectively) across all three splits with the respective feature set that achieved the highest mean inter-session test accuracy, i.e., feature set 5 for subject 1 and feature set 6 for subject 2. Reported are the results where the validation set is a subset of the training set, as discussed in the text. Non-empty cells are shaded from white (zero occurrences) to black (maximal number of occurrences).

Figure 5 shows the magnitude and phase delta radar spectrograms for the examplary word “wohnhaus” (/voːnhaʊs/) for two different repetitions, along with the frame-aligned audio spectrogram of the first repetition. This example is representative for most of the radar spectrograms and shows their similarity between repetitions. The classification results for the multi-session and inter-session evaluation for all six different sets of input features are displayed in Fig. 6. The confusion matrices for the classification results on the best-performing feature sets (i.e., set 5 and 6 for subject 1 and 2, respectively) for both subjects are displayed in Fig. 7, along with the hyperparameters of the respective BiLSTM in Table 2. With respect to the inter-session classification results, using $S_2(f)$ as input features yielded significantly higher recognition accuracies compared to $S_1(f)$. Indeed, including $S_1(f)$ into any feature set for subject 1 actually had an adversarial effect, worsening the results drastically, whereas for subject 2, it showed only very minor improvements at best.

In contrast, including both magnitude and phase delta features for $S_2(f)$ improved the mean inter-session test accuracy for subject 1 by roughly absolute 10% over the next best performing feature set and also led to a small improvements for subject 2. Most of these findings also apply to the multi-session test accuracy results, which reached almost perfect classification with $\ge$ 98% for both subjects.

**Table 2:** BiLSTM hyperparameters for each of the networks that produced the confusion matrices in Fig. 7.

	Feature set nr.	# Input features	# Hidden units	Batch size	Learning rate	# Epochs
**Multi-session**						
Subject 1	5	201	38	8	0.00164	47
Subject 2	6	268	73	8	0.00078	112
**Inter-session**						
Subject 1						
Split 1	5	201	76	8	0.00160	47
Split 2	5	201	77	8	0.00136	41
Split 3	5	201	44	8	0.00202	61
Subject 2						
Split 1	6	268	97	8	0.00076	45
Split 2	6	268	83	8	0.00105	72
Split 3	6	268	88	8	0.00132	70

Only a single BiLSTM layer was used.

## Discussion

Whereas SFCW radar can be slower, compared to, e.g., impulse radar, it also has the advantage of precise frequency selection. This is advantageous for regulatory reasons and for selecting the frequency band(s) which yield the best results for the required task. Although we did investigations on the optimal set of frequency components for the transmission spectra^34^, this is still rather unexplored and additionally dependent on the type of antenna or classifier architecture used. As such, being able to alter the frequency content of the transmission spectra almost freely is very beneficial. A general observation was that the transmission spectra were substantially less attenuated towards lower frequencies in the 1-2 GHz range (see Fig. 4). Whereas this trend might extend to even lower frequencies, there are also practical and physical antenna size constraints. Sizes above a few centimeters were found impractical, because the antennas were no longer in good contact with the skin (unless fabricated on a flexible substrate, which has its own caveats).

We initially experimented with the inclusion of reflection spectra in addition to the transmission spectra, but found that they were substantially more susceptible to antenna placement & movement and ultimately decided against using them. Head size also had an influence on the spectral dynamics as it influenced the distance between the three antennas. A larger head resulted in less spectral dynamics, which can be seen in Fig. 4 on subject 2. This also led to less distinct delta features, which is most likely the reason why they had less impact on the test classification results for subject 2.

As for the input feature selection presented in this work, we deliberately used straightforward transformations (magnitude, phase and delta features) to establish a baseline for the recognition accuracy in both the multi-session and inter-session case for our proposed measuring technique. This leaves a lot of room for improvement on the already very promising results, especially for the inter-session dependency.

BiLSTMs as a classifier were chosen because the radar spectrograms followed a temporal order which also varied in length (42 to 113 frames across all 3000 sequences). This presented a standard case for RNNs, especially LSTMs, which eliminate several shortcomings of classical RNNs for longer sequences^39^. The bidirectional type was chosen, because feeding the network with the sequence from both directions adds temporal context not present in the unidirectional case and can increase training speed^40^, as was observed in our experiments. There also exist other types of RNNs (including their bidirectional variants), such as RNNs with gated recurrent units (similar to LSTMs), recursive neural networks or echo state networks^39^. Additionally, other network architectures such as convolutional neural networks (CNNs and temporal CNNs)^39^, Transformer^41^ or Conformer networks^42^ can be used for sequence-to-label tasks and the attention mechanism also applied to RNNs^43^. However, because BiLSTMs already worked very well and are a somewhat standard classifier for sequence-to-label tasks, we refrained from testing other classifier for this particular study for reasons of conciseness.

Initial experiments with a 5-fold cross-validation during training showed that the partition noise of the validation accuracy within the training set was in the range of only a few percent for both subjects and that the validation accuracies reached above 98% regularly. As a result, there was little “room” to differentiate the optimal hyperparameter set from suboptimal ones and the highest validation accuracy often did not reveal the optimal hyperparameter set (i.e., the one associated with the highest test accuracy). This discrepancy was on the order of 1–7% for well performing and as high as 30% for the worst performing feature sets (the ones including $S_1(f)$ for subject 1). Using a small fraction of the test set as a set-aside validation set substantially improved the hyperparameter search and revealed the optimal ones in almost all cases, leading to higher test accuracies and less variability between session splits (Fig. 6). In a model deployment scenario, this would necessitate retraining, once a small set of utterances becomes available for the current session, which is rather undesirable. This problem might be alleviated with the availability of more recorded data and will be investigated in further experiments.

Table 3 lists a comparison with reported isolated command word recognition results across different SSI technologies for session-dependent/multi-session and inter-session evaluations (if available). Note that “session-dependency” strictly speaking refers to training and testing on a *single* session only^35^, but was merged in Table 3 with multi-session results, because they can be interpreted very similarly. Entries marked with a dagger superscript$^{\dagger }$ also reported phoneme recognition rates, which were omitted in this comparison. This also applies to Kim *et al.*^12^, who actually reported *speaker*-independent (i.e., cross-speaker) phoneme error rates on healthy and laryngectomy patients using EMA. Similarly, Stone *et al.*^16^ were the first to report intra-speaker and cross-speaker recognition rates on a small corpus of 10/30 isolated words (digits and German command words) with recognition accuracies of 99.5%/97% and 61.75%/56.17%, respectively, using EOS. Whereas our study explored the performance of the proposed radar system for the recognition of isolated words within and across sessions of the same speaker (including the associated variability of word pronunciation and antenna placement) the assessment of cross-speaker performance (with its variability due to anatomical differences) is a step up in difficulty and remains to be investigated in future studies. Additionally, although we did not conduct a systematic noise analysis, these sources of “physiological” noise are expected to be substantially higher for the selected frequency band (with antenna placement being the most relevant, followed by pronunciation style) compared to internal system noise or noise caused by interfering signals in an everyday environment.

Table 3 also distinguishes, whether the speech data was collected vocalized or subvocally (also termed *mouthed speech*), as there exist discrepancies between both modalities^44^, which have an impact on the recognition (or synthesis) task. This will be another subject of investigation in the future as our results currently apply to vocalized recordings.

According to Table 3, data on inter-session dependency for SSIs is very sparse and most work so far has focused on speaker-dependent, single or multi-session experiments. To the best of our knowledge, only sEMG, as a fully non-invasive SSI modality, has been evaluated for inter-session dependencies. sEMG currently also leads the field, especially for large vocabulary^5,35^. However, given our presented results, there are a number of compelling reasons for RBS as an alternative: RBS is also fully non-invasive and, when compared with other small-vocabulary word recognition results, yields state of the art results on both multi-session and inter-session recognition tasks. Whereas there are physical constraints on the antenna size, the remaining hardware can potentially be miniaturized and, e.g., integrated into some form of headset, similar to conventional headphones. Additionally, a pair of RX/TX antennas has a much richer “sensing profile”, compared to two sEMG electrodes, since the resulting transmission spectra apparently capture (part of) the physical state of the vocal tract.

**Table 3:** Comparison between isolated word recognition results across different SSI technologies.

Modality	Dictionary	Session-dependent or multi-session accuracy (%)	Inter-session accuracy (%)	Classifier	Vocalization
PMA^4^	9 words$^{\dagger }$	94	–	DTW-(kNN)	Vocalized
PMA^7^	57 words	98.8	–	GMM-HMM	Vocalized
EPG^13^	21 words	84.36	–	DTW-GAK	Vocalized
EMA^11^	25 words$^{\dagger }$	96.88	–	SVM	Vocalized
sEMG^21^	65 words	90.4	–	GMM-HMM	Subvocal
sEMG^35^	108/2102 words	89.55/67	78.06/49.52	GMM-HMM	Training vocalized, test subvocal
sEMG^5^	2500 words	89.7	–	GMM-HMM	Subvocal
sEMG^22^	110 words	92.1	–	LDA	Subvocal
RBS^29^	10 words	92.8	–	Template matching	Vocalized
RBS (Our work)	50 words	99.17	88.87	BiLSTM	Vocalized

For example, the two label–prediction pairs (taken from Fig. 7) that were confused by far the most often were “Mühe” (/myːə/) – “Höhe” (/høːə/) and “Zeugin” (/ʦɔʏgɪn/) – “Neun” (/nɔʏn/), both of which are phonetically similar. Whereas this does not apply to all confusions (e.g., the third highest mismatch was “Akku” (/akʊ/) – “Locher” (/lɔxɐ/)), it is yet another confirmation that similar phonemes tend to have similar transmission spectra (as was already demonstrated in^31^). As such, a fully phoneme-based speech recognizer will be one of the next steps in our future development.

## Data Availability

The data corpus is provided unter https://www.vocaltractlab.de/index.php?page=birkholz-supplements.

## Code availability

The code for the BiLSTM evaluation is accessible under https://github.com/TUD-STKS/radar_based_command_word_recognition.

## References

[1] Gonzalez-Lopez JA (2020). Silent speech interfaces for speech restoration: A review. IEEE Access.

[2] Schultz T (2017). Biosignal-based spoken communication: A survey. IEEE/ACM Trans. Audio Speech Lang. Process..

[3] Denby B (2010). Silent speech interfaces. Speech Commun..

[4] Fagan MJ, Ell SR, Gilbert JM, Sarrazin E, Chapman PM (2008). Development of a (silent) speech recognition system for patients following laryngectomy. Med. Eng. Phys..

[5] Meltzner GS (2017). Silent speech recognition as an alternative communication device for persons with laryngectomy. IEEE/ACM Trans. Audio Speech Lang. Process..

[6] Gilbert J (2010). Isolated word recognition of silent speech using magnetic implants and sensors. Med. Eng. Phys..

[7] Hofe R (2013). Small-vocabulary speech recognition using a silent speech interface based on magnetic sensing. Speech Commun..

[8] Gonzalez JA (2016). A silent speech system based on permanent magnet articulography and direct synthesis. Comput. Speech Lang..

[9] Wrench, A. A. & Richmond, K. Continuous speech recognition using articulatory data. In *Proceedings of 6th International Conference on Spoken Language Processing (ICSLP)*, 1–4 (2000).

[10] Wang J, Green JR, Samal A, Yunusova Y (2013). Articulatory distinctiveness of vowels and consonants: A data-driven approach. J. Speech Lang. Hear. Res..

[11] Wang J, Samal A, Rong P, Green JR (2016). An optimal set of flesh points on tongue and lips for speech-movement classification. J. Speech Lang. Hear. Res..

[12] Kim M, Cao B, Mau T, Wang J (2017). Speaker-independent silent speech recognition from flesh-point articulatory movements using an LSTM neural network. IEEE/ACM Trans. Audio Speech Lang. Process..

[13] Li, R., Wu, J. & Starner, T. Tongueboard: An oral interface for subtle input. In *Proceedings of 10th Augmented Human International Conference (AH)*, 1–9, 10.1145/3311823.3311831 (2019).

[14] Zin SM, Rasib SZM, Suhaimi FM, Mariatti M (2021). The technology of tongue and hard palate contact detection: A review. Biomed. Eng. Online.

[15] Stone, S. & Birkholz, P. Silent-speech command word recognition using electro-optical stomatography. In *Proceedings of 17th Annual Conference of the International Speech Communication Association (Interspeech)*, 2350–2351, 10.1109/ICASSP40776.2020.9053447 (2016).

[16] Stone, S. & Birkholz, P. Cross-speaker silent-speech command word recognition using electro-optical stomatography. In *Proceedings of 45th International Conference on Acoustics, Speech, and Signal Processing (ICASSP)*, 7849–7853, 10.1109/ICASSP40776.2020.9053447 (2020).

[17] Wagner C (2022). Evaluation of a non-personalized optopalatographic device for prospective use in functional post-stroke dysphagia therapy. IEEE Trans. Biomed. Eng..

[18] Betts BJ, Binsted K, Jorgensen C (2006). Small-vocabulary speech recognition using surface electromyography. Interact. Comput..

[19] Lee K-S (2008). EMG-based speech recognition using hidden Markov models with global control variables. IEEE Trans. Biomed. Eng..

[20] Wand, M. & Schultz, T. Towards real-life application of EMG-based speech recognition by using unsupervised adaptation. In *Proceedings of 15th Annual Conference of the International Speech Communication Association (Interspeech)*, 10.21437/Interspeech.2014-301 (2014).

[21] Meltzner GS (2018). Development of sEMG sensors and algorithms for silent speech recognition. J. Neural Eng..

[22] Wang Y (2021). All-weather, natural silent speech recognition via machine-learning-assisted tattoo-like electronics. NPJ Flex. Electron..

[23] Toth, A. R., Kalgaonkar, K., Raj, B. & Ezzat, T. Synthesizing speech from Doppler signals. In *Proceedings of 35th IEEE International Conference on Acoustics, Speech and Signal Processing (ICASSP)*, 4638–4641, 10.1109/ICASSP.2010.5495552 (2010).

[24] Csapó, T. G., Grósz, T., Gosztolya, G., Tóth, L. & Markó, A. DNN-based ultrasound-to-speech conversion for a silent speech interface. In *Proceedings of 18th Annual Conference of the International Speech Communication Association (Interspeech)*, 3672–3676, 10.21437/Interspeech.2017-939 (2017).

[25] Wand, M., Koutník, J. & Schmidhuber, J. Lipreading with long short-term memory. *Proceedings of 41st IEEE International Conference on Acoustics, Speech and Signal Processing (ICASSP)* 6115–6119, 10.1109/ICASSP.2016.7472852 (2016).

[26] Shillingford, B. *et al.* Large-scale visual speech recognition. ArXiv preprint arXiv:1807.05162 (2018).

[27] Hueber T (2010). Development of a silent speech interface driven by ultrasound and optical images of the tongue and lips. Speech Commun..

[28] Holzrichter JF, Burnett GC, Ng LC, Lea WA (1998). Speech articulator measurements using low power EM-wave sensors. J. Acoust. Soc. Am..

[29] Eid AM, Wallace JW (2009). Ultrawideband speech sensing. IEEE Antennas Wirel. Propag. Lett..

[30] Shin YH, Seo J (2016). Towards contactless silent speech recognition based on detection of active and visible articulators using IR-UWB radar. Sensors.

[31] Birkholz P, Stone S, Wolf K, Plettemeier D (2018). Non-invasive silent phoneme recognition using microwave signals. IEEE/ACM Trans. Audio Speech Lang. Process..

[32] Geiger, M., Schlotthauer, D. & Waldschmidt, C. Improved throat vibration sensing with a flexible 160-GHz radar through harmonic generation. In *Proceedinsg of IEEE/MTT-S International Microwave Symposium (IMS)*, 123–126, 10.1109/MWSYM.2018.8439458 (2018).

[33] Wen, L., Gu, C. & Mao, J. -F. Silent speech recognition based on short-range millimeter-wave sensing. In *Proceedings of IEEE/MTT-S International Microwave Symposium (IMS)*, 779–782, 10.1109/IMS30576.2020.9223988 (2020).

[34] Digehsara, P. A. *et al.* On the optimal set of features and the robustness of classifiers in radar-based silent phoneme recognition. In *Studientexte zur Sprachkommunikation: Elektronische Sprachsignalverarbeitung 2021*, (eds. Hillmann, S., Weiss, B., Michael, T. & Möller, S.) 112–119 (TUDPress, 2021).

[35] Wand, M. & Schultz, T. Session-independent EMG-based speech recognition. In *Proceedings of 4th International Conference on Bio-inspired Systems and Signal Processing*, 295–300, 10.1109/ASRU.2005.1566521 (Italy, Rome, 2011).

[36] Holzrichter, J. F. Characterizing silent and pseudo-silent speech using radar-like sensors. In *Proceedings of 10th Annual Conference of the International Speech Communication Association (Interspeech)*, 628–631, 10.21437/Interspeech.2009-223 (2009).

[37] Paszke, A. *et al.* Pytorch: An imperative style, high-performance deep learning library. In Wallach, H. *et al.* (eds.) *Proceedings of 32nd Conference on Neural Information Processing Systems*, 8024–8035 (Curran Associates, Inc., 2019).

[38] Maier-Hein, L., Metze, F., Schultz, T. & Waibel, A. Session independent non-audible speech recognition using surface electromyography. In *Proceedings of IEEE Workshop on Automatic Speech Recognition and Understanding, 2005*, 331–336, 10.1109/ASRU.2005.1566521 (2005).

[39] Goodfellow I, Bengio Y, Courville A (2016). Deep Learning.

[40] Graves A, Schmidhuber J (2005). Framewise phoneme classification with bidirectional LSTM and other neural network architectures. Neural Netw.

[41] Vaswani, A. *et al.* Attention is all you need. ArXiv preprint arXiv:1706.03762v5 (2017).

[42] Gulati, A. *et al.* Conformer: Convolution-augmented transformer for speech recognition. ArXiv preprint arXiv:2005.08100 (2020).

[43] Bahdanau, D., Cho, K. & Bengio, Y. Neural machine translation by jointly learning to align and translate. ArXiv preprint arXiv:1409.0473 (2014).

[44] Wand M, Janke M, Schultz T (2014). Tackling speaking mode varieties in EMG-based speech recognition. IEEE Trans. Biomed. Eng..

